# Gut DNA Virome Diversity and Its Association with Host Bacteria Regulate Inflammatory Phenotype and Neuronal Immunotoxicity in Experimental Gulf War Illness

**DOI:** 10.3390/v11100968

**Published:** 2019-10-21

**Authors:** Ratanesh K. Seth, Rabia Maqsood, Ayan Mondal, Dipro Bose, Diana Kimono, LaRinda A. Holland, Patricia Janulewicz Lloyd, Nancy Klimas, Ronnie D. Horner, Kimberly Sullivan, Efrem S. Lim, Saurabh Chatterjee

**Affiliations:** 1 Environmental Health and Disease Laboratory, Department of Environmental Health Sciences, University of South Carolina, Columbia, SC 29208, USA; sethr@mailbox.sc.edu (R.K.S.); mondola@mailbox.sc.edu (A.M.);; 2 Center for Fundamental and Applied Microbiomics, The Biodesign Institute, Arizona State University, Tempe, AZ 85281, USA; 3 School of Public Health, Boston University, Boston MA 02118, USA; paj@bu.edu (P.J.L.); tty@bu.edu (K.S.); 4 NOVA Southeastern University, Fort Lauderdale, FL 33314, USA; nklimas@nova.edu; 5 Department of Health Services Policy and Management, University of South Carolina, Columbia, SC 29208, USA; hornerrd@mailbox.sc.edu; 6 Dorn VA Medical Center, Columbia, SC 29209, USA

**Keywords:** Gulf war illness, Virome, microbiome, next-generation sequencing, intestinal inflammation, neuroinflammation, Ribavirin, IL6

## Abstract

Gulf War illness (GWI) is characterized by the persistence of inflammatory bowel disease, chronic fatigue, neuroinflammation, headache, cognitive impairment, and other medically unexplained conditions. Results using a murine model show that enteric viral populations especially bacteriophages were altered in GWI. The increased viral richness and alpha diversity correlated positively with gut bacterial dysbiosis and proinflammatory cytokines. Altered virome signature in GWI mice also had a concomitant weakening of intestinal epithelial tight junctions with a significant increase in Claudin-2 protein expression and decrease in ZO1 and Occludin mRNA expression. The altered virome signature in GWI, decreased tight junction protein level was followed by the presence an activation of innate immune responses such as increased Toll-like receptor (TLR) signaling pathways. The altered virome diversity had a positive correlation with serum IL-6, IL-1β, and IFN-γ, intestinal inflammation (IFN-γ), and decreased Brain-Derived Neurotrophic Factor (BDNF), a neurogenesis marker. The co-exposure of Gulf War chemical and antibiotic (for gut sterility) or Gulf War chemical and Ribavirin, an antiviral compound to suppress virus alteration in the gut showed significant improvement in epithelial tight junction protein, decreased intestinal-, systemic-, and neuroinflammation. These results showed that the observed enteric viral dysbiosis could activate enteric viral particle-induced innate immune response in GWI and could be a novel therapeutic target in GWI.

## 1. Introduction

Gulf war illness (GWI) is a chronic multisymptomatic, medically unexplained disorder that affected 25%–33% of the veterans returned from the Persian Gulf War in 1990–1991 [1]. Gulf war illness is a wide range of acute and chronic illnesses characterized by gastrointestinal (GI) discomforts (eg, inflammatory bowel disease (IBD)), muscle pain, joint pain, chronic fatigue, headache, and cognitive impairment [2,3]. Although the exact cause of these illnesses is unknown, recent research has found an association between environmental exposures in the Gulf war theatre and symptoms presented by sufferers [4,5]. During the war, veterans were exposed to several chemicals including sarin nerve gas, pyridostigmine bromide (PB) anti-nerve gas pills, insecticides, and insect repellents (permethrin) [1,6]. In a study by Koo et al. mimicking physical and mental stress by corticosterone along with war theater toxicants resulted in enhanced neuroimmune disorder and inflammatory phenotype, in an animal model of GWI [7]. In other studies, mice treated with PB, pesticides, and permethrin (insect repellent) showed cognitive impairment, learning difficulty, fatigue and GI dysfunction [8,9]. Similarly, our recent report, we have shown that mice exposed to PB and permethrin along with stress hormone corticosterone causes gut microbiome alteration, endotoxemia and intestinal and neuroinflammation [4,10]. However, how these gut bacterial communities are regulated in GWI is largely unknown. This forms the basis of our present study.

The gut harbors a complex microbiome community of bacteria, viruses, protozoans, fungi, and other microorganisms that can impact health and disease [11]. Maintenance of balanced homeostatic interactions is important for host immune functions [12]. Conversely, alterations in host-microbiota interactions lead to immune modulation, metabolism regulation and neuroendocrine response in specific or systemic inflammatory disease phenotypes [13,14,15,16]. With the advent of next-generation sequencing (NGS), metagenomics studies revealed that the healthy microbial composition imparts phenotypic differences between individuals, helps in complex food digestion and absorption, maintains gut barrier integrity function, prevents pathogenic microbial-host interaction, and regulates host immune system [17,18]. Recent reports also suggested that the gut microbial dysbiosis or alteration in a healthy gut microbial communities is associated with several diseases such as obesity, type 2 diabetes (T2D), inflammatory bowel disease (IBD), atherosclerosis, allergy, colon cancer, and cognitive behavior [19,20,21,22,23,24,25]. However, the role of gut microbiota in a Gulf war illness phenotype is poorly understood.

The enteric virome, which includes eukaryotic viruses and prokaryotic viruses (bacteriophages), is also implicated in gut inflammation and immunity [26,27]. Alterations in the gut virome and expansion in *Caudovirales* bacteriophages are associated with Crohn’s disease and ulcerative colitis [28]. The bacteriophages regulate gut bacterial composition, community structure, and function, and thus immune responses indirectly [29]. We and others have recently shown that the altered gut bacterial community is associated with intestinal inflammation, neuroinflammation, myalgic encephalomyelitis, chronic fatigue syndrome, metabolic syndrome, and Gulf War illness (GWI) [4,10,22,30,31]. In GWI, the altered gut bacteriome induced by Gulf War chemical exposure follows Toll-like receptor 4 (TLR4) mediated pathway leading to intestinal and neuroinflammation. Since the gut bacterial community is hugely regulated by the bacteriophages, therefore, it paved the way for newer investigation of virome dysbiosis and how this alteration leads to TLR-mediated inflammatory surge in the intestine and frontal cortex in GWI. 

In this study, we examine the effect of Gulf War chemical exposure on the gut virome and bacterial microbiome in a well-established mouse model of Gulf war illness. Importantly, here we unravel the TLR-mediated inflammatory pathways induced by enteric viral and bacterial PAMPs (pathogen-associated molecular pattern). We also test the hypothesis that the use of antibiotics and antiviral restores bacterial community and viral community respectively to the healthy state and ameliorate inflammatory surge in GWI.

## 2. Materials and Methods

### 2.1. Materials

We purchased pyridostigmine bromide (PB), permethrin (Per), Neomycin trisulfate hydrate, Enrofloxacin, and Ribavirin from Sigma-Aldrich (St. Louis, MO, USA). Anti-claudin-2, anti-MyD88, anti-MCP-1, and anti-β-actin primary antibodies were purchased from Abcam (Cambridge, MA, USA). anti-β-tubulin, anti-TLR3, anti-TLR7, anti-IKKα, anti-p65, and anti-IL6 primary antibodies were purchased from Santacruz Biotechnology (Dallas, TX, USA) while anti-TRAF6 was purchased from Abclonal Technology (Woburn, MA, USA). Species-specific biotinylated conjugated secondary antibodies and Streptavidin-HRP (Vectastain Elite ABC kit) were purchased from Vector Laboratories (Burlingame, CA, USA). Fluorescence-conjugated (Alexa Fluor) secondary antibodies and ProLong Diamond antifade mounting media with DAPI were purchased from Thermofisher Scientific (Grand Island, NY, USA). All other chemicals used in this study were purchased from Sigma unless otherwise specified. Animal tissues were paraffin-embedded and sectioned into slides by AML laboratories (Baltimore, MD, USA).

### 2.2. Animals

Adult wild-type male (C57BL/6J mice, 10 weeks old) were purchased from Jackson Laboratories (Bar Harbor, ME). Mice were implemented in accordance with NIH guideline for human care and use of laboratory animals and local IACUC standards. All procedures were approved by the University of South Carolina at Columbia, SC. Mice were housed individually and fed with a chow diet at 22–24 °C with a 12-h light/12-h dark cycle. All mice were sacrificed after animal experiments had been completed in one week. Serum was prepared from the blood that had been freshly obtained from mice immediately after anesthesia, by cardiac puncture. It was then preserved at −80 °C until needed for analysis. The mice were dissected, and the frontal cortex and distal parts of the small intestines were collected, fixed in Bouin’s (Sigma-Aldrich, St. Louis MO, USA) solution or 10% neutral buffered formaldehyde respectively and further processed for immunostaining and visualizations. Finally, fecal pellets and luminal contents were collected for microbiome and virome analysis.

### 2.3. Rodent Model of Gulf War Illness (GWI)

We exposed the mice to Gulf War chemicals (permethrin and pyridostigmine bromide) following well-established rodent models of Gulf War illness [32,33]. The mice were randomly divided into four groups and were administered with four doses of vehicle to the control mice and GW chemicals to the GWI mice by oral gavage. The first group that is controls (*n* = 11) were treated with vehicle (6% DMSO diluted in PBS). The second group is GWI (treated group; *n* = 11), mice received permethrin (200 mg/Kg, diluted in DMSO and PBS; the final concentration of DMSO was 6%) and pyridostigmine bromide (2 mg/Kg diluted in PBS) by oral gavage. The third group is GW chemicals and antibiotics (GWI+AB) (*n* = 11), mice were exposed to permethrin and PB as group 2 and also exposed to antibiotics (Enrofloxacin 1 mg/Kg in DMSO and neomycin 45 mg/Kg in PBS, final concentration of DMSO was 6%) by oral gavage and the fourth group is GW chemical and antiviral (Ribavirin) group-(GWI+AV) (*n* = 11), mice were treated with permethrin and PB as group 2 and also with broad-spectrum antiviral drug Ribavirin (100 mg/Kg, PBS) every day for 7 days by IP injections for partial depletion of the gut virome. This antiviral group was essential to show an association with the virome and GWI pathology if any.

### 2.4. Microbiome Analysis

#### Virus-Like Particle (VLP) Enrichment and Total Nucleic Acid Extraction

VLPs were enriched from mouse stool using a protocol as previously described [28]. Mouse fecal specimens (control *n* = 11; GWI *n* = 11; GWI+AB *n* = 10; GWI+AV *n* = 11) were randomized using random number generator. Mouse fecal specimens (approximately 200 mg) were diluted in SM Buffer (G-Biosciences) in a 1:6 ratio, vortexed at 3000 rpm for 10 min, then centrifuged at 7000× *g* for 10 min. The supernatant was filtered through a 0.45 μm membrane (Celltreat). The stool filtrates were treated with lysozyme (Sigma) and baseline-ZERO DNase (Lucigen) to degrade unencapsidated DNA. DNase treatment was inactivated with EDTA (5mM final concentration). Total nucleic acid was extracted using the eMAG instrument (bioMérieux). Negative controls were included in the same extraction process to assess contamination. Controls spiked with Enterobacteria phage λ DNA were used to assess cross-contamination and amplification. VLP DNA was amplified with GenomiPhi V2 (GE Healthcare) before Illumina library construction. Samples and negative controls were pooled for sequencing in three Illumina MiSeq v2 2 × 250 bp sequencing runs (Supplementary Figure S1). 

### 2.5. Virome Analysis

Illumina sequencing reads (2 × 250) (averaged 1.19 ± 0.48 million reads per sample) were quality filtered to remove adapters and low-quality bases using BBTools (Bushnell B.– sourceforge.net/projects/bbmap/). High quality-filtered reads were queried against viral RefSeq + Neighboring sequences database using BLASTx to identify viral reads. The taxonomic lineage of viral reads was assigned using MEGAN (version 6.15.2) naïve LCA algorithm with the parameters min support = 1, min support percentage = 0, and bitscore of 100 [34]. 

Contamination and run-to-run bias effects were assessed in the following ways: 1) There were no significant differences in the sequencing depth between the three Illumina runs or between sample types (Supplementary Figure S1A,B); 2) PCoA analysis of the unweighted Bray–Curtis distances showed that the viromes were not influenced by Illumina sequencing run (Supplementary Figure S1C); 3) negative controls were distinct from mouse fecal specimens (Supplementary Figure S1C); 4) Enterobacteria phage λ (spiked control) were only found in PBS controls and not in mouse specimens (Supplementary Figure S1D). In order to better identify the divergent phages contig analyses of the virome is also provided in Supplementary Figure S1L–N. Viral contigs were assembled from quality-filtered reads individually for each sample using IDBA-UD (Version 1.1.0) [35]. Next, contigs from all samples were filtered by length (greater than 500 bp), de-duplicated using BBTools (minidentity ≥ 99), and overlapping contigs merged using Minimus2 [36]. Subsequently, the contigs were processed through VirSorter to identify viral contigs [37]. VirSorted called viral contigs were collated into a database, quality filtered reads of each sample were then mapped against the contig database using BWA-MEM (parameters -L 97, 97, -M) [38]. 

### 2.6. Bacteriome Analysis

DNA isolation, sequencing, and analysis of gut microbiome were done at CosmosID (Rockville, MD, USA) using vendor optimized protocol. Briefly, DNA was isolated from mouse fecal samples using the ZymoBIOMICS Miniprep kit, following the manufacturer’s instructions. CosmosID’s optimized 16S sequencing was carried out which covers the V3-V4 (341 nt–805 nt) region of the 16S rRNA gene with a two-step PCR strategy. The first step is to perform PCR using 16S-optimized primer set to amplify the V3–V4 regions of 16S rDNA within the metagenomic DNA. Next, the PCR products from the previous steps are mixed at equal amounts and used as templates in the second step to produce Illumina dual-index libraries for sequencing, with both adapters containing an 8-bp index allowing for multiplexing. The dual-indexed library amplification products are purified using Ampure beads (Beckman Coulter). Library quantification is performed using Qubit dsDNA HS assay (ThermoFisher) and qualified on a 2100 Bioanalyzer instrument (Agilent) to show a distribution with a peak in the expected range. A final qPCR quantification was performed before loading onto a MiSeq (Illumina) sequencer for PE250 (v2 chemistry). The sequences for each sample were then run on the 16S pipeline of the CosmosID GENIUS software, and results were analyzed. 

Both virome and bacteriome ecological analyses (richness, alpha diversity—Shannon Index) were performed using Vegan R package (version 2.5-2) [39]. Bray–Curtis distance (beta diversity) was calculated from an unweighted presence-absence species data matrix using QIIME and visualized in R [40]. Statistical significance was assessed using a non-parametric Mann–Whitney *U* test. 

Transkingdom interactions were assessed by calculating Pearson’s correlation coefficients between bacteria and viruses using the R package gplots (Figure 5E) and Originlab2019b package (Supplementary Figure S2). Hierarchical clustering was performed on the control group, and the same clustering order was used to visualize correlations of the other datasets (GWI, GWI+AB, and GWI+AV). 

### 2.7. Availability of Data and Materials

Sequence data will be deposited to the NCBI Sequence Read Archive under BioProject accession number PRJNA561629.

### 2.8. Immunohistochemistry 

Paraffin-embedded tissues of frontal cortex or the distal part of the small intestine were prepared according to standard protocols and sectioned to 5 µM thick. These sections were subjected to deparaffinization using a standard protocol. Epitope retrieval solution and steamer (IHC-Word, Woodstock, MD, USA) were used for epitope retrieval of the tissue sections. Three percent H_2_O_2_ was used to block the endogenous peroxidase. After serum blocking, the tissue was incubated overnight at 4.0 °C with primary antibodies against MCP1 and IL6. Species-specific biotin-conjugated secondary antibodies and streptavidin-conjugated with HRP were used to implement antigen-specific immunohistochemistry. 3,3’-Diaminobenzidine (DAB) (Sigma Aldrich, St Louis, MD, USA) was used as a chromogenic substrate. Mayer’s Hematoxylin solution (Sigma Aldrich) was used as a counterstain. Sections were washed between the steps using PBS and tween-20 (PBS-T, 1X). Finally, stained sections were mounted with Aqua mount (Lerner Laboratories, Kalamazoo, MI, USA). Tissue sections were observed using an Olympus BX51 microscope (Olympus, America). Cellsens software from Olympus America (Center Valley, PA, USA) was used for morphometric analysis of images.

### 2.9. Immunofluorescence Staining

The paraffin-embedded distal part of the small intestine or frontal cortex were deparaffinized using a standard protocol. Epitope retrieval solution and steamer were used for epitope retrieval of sections. Primary antibodies such as TLR3, TLR7, TLR9, MyD88, β tubulin, TRAF6, Claudin-2, CD40, and CD11b were used at the recommended dilution. Species-specific secondary antibodies conjugated with Alexa Fluor (633-red and 488-green) were used at recommended dilution. The stained sections were then mounted using Prolong Diamond antifade reagent with DAPI. Sections were observed under Olympus fluorescence microscope using 20×, or 40× objective lenses. Cellsens software from Olympus America (Center Valley, PA, USA) was used for morphometric analysis of images.

### 2.10. Real-Time Quantitative PCR

mRNA expression in the small intestine and frontal cortex was examined by quantitative real-time PCR analysis. Total RNA was isolated by tissue homogenization in TRIzol reagent (Invitrogen, Carlsbad, CA, USA) according to the manufacturer’s instructions and purified with the use of RNeasy mini kit columns (Qiagen, Valencia, CA, USA). cDNA was synthesized from purified RNA (1 µg) using iScript cDNA synthesis kit (Bio-rad, Hercules, CA, USA) following the manufacturer’s standard protocol. Real-time qPCR (qRTPCR) was performed with the gene-specific primers using SsoAdvanced SYBR Green Supermix and CFX96 thermal cycler (Bio-rad, Hercules, CA, USA). Threshold cycle (Ct) values for the selected genes were normalized against respective samples internal control 18S. Each reaction was carried out in triplicates for each gene and for each sample. The relative fold-change was calculated by the 2−∆∆Ct method. The sequences for the primers used for real-time PCR are provided in Table 1.

### 2.11. Western Blot

30 µg of denatured mouse small intestine and frontal cortex protein was loaded per well on Novex 4%–12% bis-tris gradient gel and subjected to standard SDS-PAGE. Separated protein bands were transferred to nitrocellulose membrane using the Trans-Blot Turbo transfer system (Bio-rad, Hercules, CA). After Ponceau S staining then the membrane was blocked with 5% non-fat milk solution for 1 h and then incubated with primary antibodies for overnight at 4 °C. A species-specific anti-IgG secondary antibody conjugated with HRP was used to tag the primary antibody. ECL Western blotting substrate was used to develop the blot. Finally, the blot was imaged using G: Box Chemi XX6 and subjected to densitometry analysis using Image J software.

### 2.12. Serum ELISA 

Serum IL6 and IFNβ, IFNγ was estimated using an ELISA kit from ProteinTech (Rosemont, IL, USA) following manufacturer protocol.

### 2.13. Statistical Analysis 

Prior to initiation of the study, we conducted calculations for each experimental condition with appropriate preliminary data to confirm that the sample number is sufficient to achieve a minimum statistical power of 0.80 at an alpha of 0.05. We used a total of 11 mice for both the control group and GW treated groups; 9 mice for both GW+AB and GW+AV treated mice. Data from each group were pooled. Student’s *t*-test was used to compare means between two groups at the termination of treatment. A one-way ANOVA was applied as needed, to evaluate differences among treatment groups followed by the Bonferroni post-hoc correction for intergroup comparisons. Association was estimated by performing a Pearsons Rank Product moment coefficient analysis.

## 3. Results 

### 3.1. Gulf War Chemical Exposure Alters the Gut Virome

**Figure 1:**
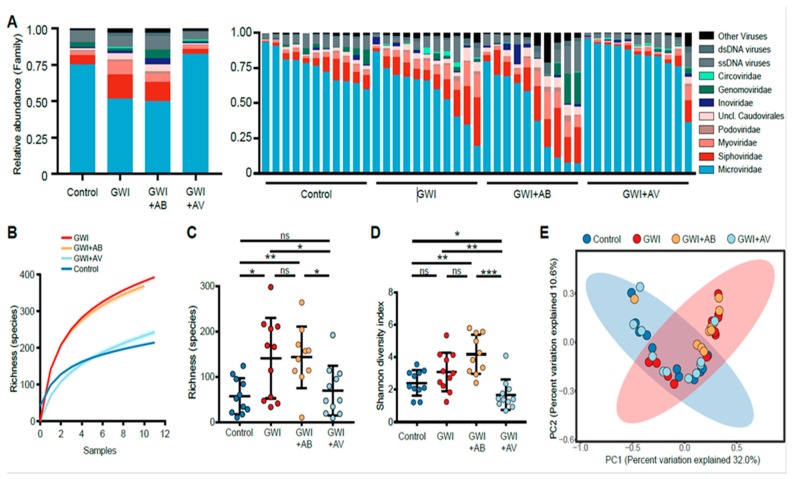
Virome alterations in a mouse model of Gulf War Illness. (**A**) The relative abundance of DNA virome is shown of the treatment group average (left) and by individual mice (right). (**B**) Rarefaction curves of virome richness are plotted with an increasing number of mice sub-samplings from each treatment group. Curves represent the average of 500 iterations at each sub-sampling depth. (**C**) Viral species richness of each group and (**D**) Shannon diversity index of virus species is shown. Statistical significance was assessed by Mann–Whitney *U* test; * *p* < 0.05, ** *p* < 0.01, *** *p* < 0.001, ns not significant. (**E**) Principal coordinate analysis (PCoA) of binary Jaccard distance of virome species. Treatment groups are indicated respectively. The 95% confidence intervals of the control group (blue) and GWI group (red) are shaded accordingly.

Exposure to Gulf War chemicals leads to profound intestinal inflammatory injury and perturbations of the gut microbiome [4]. Hence, we sought to understand the role of the gut virome in GWI. Using a mouse model of GWI, we performed next-generation sequencing to characterize the gut virome of C57BL/6J mice exposed to GWI chemicals. GW chemical-exposed mice had decreased relative abundance of *Microviridae* bacteriophages and increased relative abundance of *Siphoviridae* and *Myoviridae* bacteriophages compared to control mice (Figure 1A). Further, GW chemical exposure led to an increase in viral richness (Figure 1B) and alpha diversity (Figure 1C). 

Since GWI has been associated with bacterial dysbiosis [4], we evaluated the effects of two broad-spectrum antimicrobial interventions in GWI-induced mice: 1) a combination of Neomycin and Enrofloxacin antibiotics, and the 2) antiviral nucleoside Ribavirin. Antibiotic treatment of GW chemical-exposed mice (GWI+AB) did not significantly alter the virome as compared to GWI mice in terms of virome composition, richness and alpha diversity (Figure 1A–C). However, antiviral (Ribavirin)-treated mice exposed to GW chemicals (GWI+AV) had decreased viral richness and alpha diversity when compared to GWI mice (Figure 1B,C). With antiviral treatment, the virome of mice was altered in a manner that resulted in mice having a virome composition that was more similar to control mice that were not exposed to GW chemicals as assessed by an unweighted Bray–Curtis distance PCoA analysis (Figure 1D). These data suggest that GW chemicals exposure leads to significant virome alterations that can be reversed in part by antiviral treatment, but not by antibiotics. 

### 3.2. Gut Bacterial Dysbiosis Induced by Gulf War Chemical Exposure Is Altered by Antibiotics but Not by Antiviral Treatment

**Figure 2:**
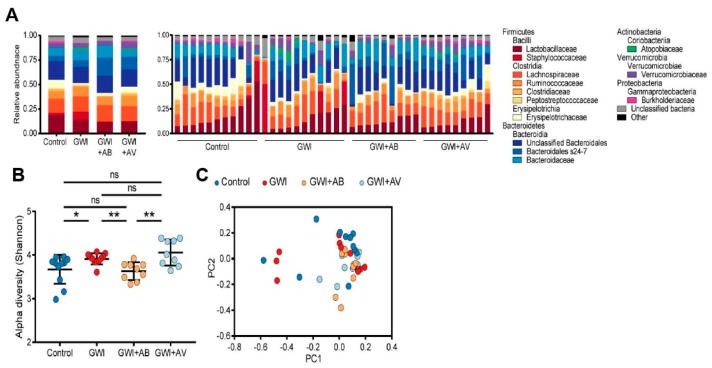
Gut bacterial dysbiosis induced by Gulf War chemical exposure is altered by antibiotics but not by antiviral treatment. (**A**) The relative abundance of bacteriome is shown of the treatment group average (left) and by individual mice (right). (**B**) Alpha diversity (Shannon index) of bacterial taxa is shown. Statistical significance was assessed by Mann–Whitney *U* test; * *p* < 0.05, ** *p* < 0.01, ns not significant. (**C**) Principal coordinate analysis (PCoA) of unweighted Bray–Curtis distance of bacteriome is shown. Treatment groups are indicated respectively.

Using the same GWI mouse model, we assessed the effects of antibiotics and antiviral treatments on gut bacterial microbiota. Consistent with our previous study, the gut bacterial microbiomes were predominantly colonized by *Firmicutes* and *Bacteroidetes* (Figure 2A). GW chemical exposure led to a significant increase in Shannon alpha diversity (Figure 2B, *p* = 0.016). Conversely, antibiotic treatment in GW-exposed mice led to decreased alpha diversity (*p* = 0.002) to levels that were not significantly different from control mice (Figure 2B, *p* = 0.261). In contrast, there was no significant difference in the bacterial microbiome alpha diversity between antiviral-treated GW mice and control mice. This suggests that while antibiotics treatment significantly altered the bacterial dysbiosis induced by Gulf War chemical exposure, antiviral treatment had minimal impact on the bacterial microbiome (Figure 2C).

**Figure 3:**
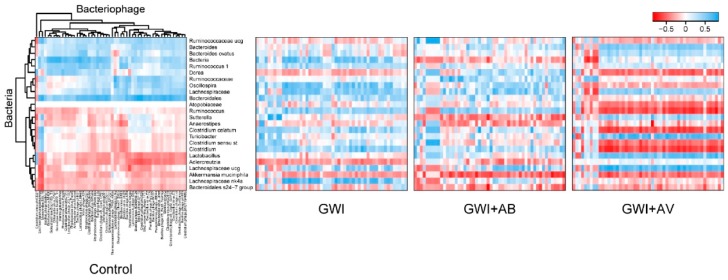
GWI and intervention alterations in bacteria-bacteriophage interactions. Heatmap of Pearson correlations between bacteria (rows) and bacteriophages (columns) are shown. Clustering was performed on the control group (left), and the same clustering order maintained in the correlation plots of Gulf War illness (GWI), GW chemicals and antibiotics (GWI+AB), and GW chemical and antiviral (Ribavirin) (GWI+AV) groups (right).

Trans-kingdom interactions between bacteria and viruses play an important role in intestinal immunity and inflammatory disease [41]. Therefore, we assessed the effects that GW chemicals and subsequent antimicrobial treatments had on transkingdom interactions between the gut bacteria and bacteriophages. In control mice, *Ruminococcaceae* and *Bacteroidia* microbiota were positively correlated with bacteriophages, while *Clostridia* and *Lactobacillaceae* microbiota were inversely correlated with bacteriophages (Figure 3). GW chemical exposure (GWI) led to significant alterations. In particular, there were more positive interactions with *Caudovirales* bacteriophages (including *Siphoviridae* and *Myoviridae*) with bacteria such as *Clostridia* and *Ruminococcaceae* species. Antibiotic treatment was associated with primarily stronger negative bacterial interactions (*Sutterella*, *Anaerostipes*, *Akkermansia*). The antiviral treatment restored specific transkingdom interactions (*Bacteroides* and *Clostridia*), but also had new strongly positive interactions between *Caudovirales* bacteriophages and *Lactobacillaceae* and *Lachnospiraceae* bacterial microbiota. Taken together, this further indicates that virome-bacterial relationships are implicated in GWI.

### 3.3. Gut Bacterial and Viral Dysbiosis Induced Alteration of Intestinal Epithelial Tight Junction Proteins Is Restored by Antibiotics and Antiviral Treatment

**Figure 4:**
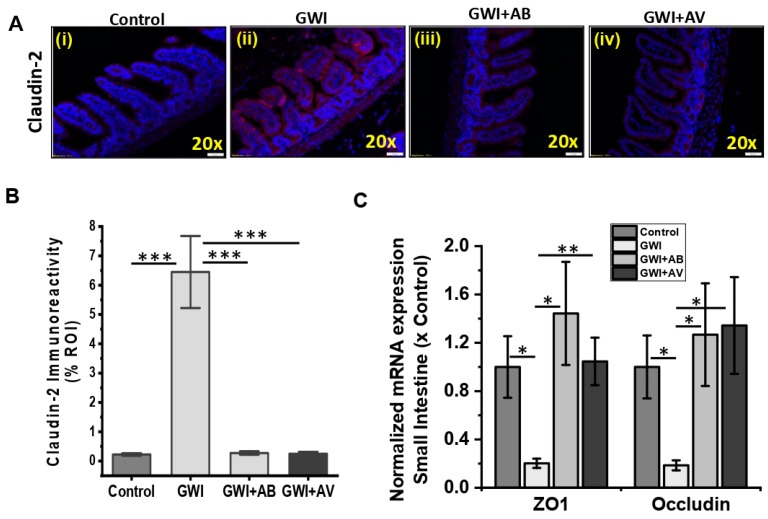
Anti-viral or antibiotic intervention improves intestinal epithelial membrane integrity and tight junctions in GWI. (**A**,**B**) Representative immunofluorescence images of small intestine showed Claudin-2 (red) expression and localization in Control, GWI (Gulf War chemical exposed mice), GWI+AB (Gulf War chemical and antibiotic exposed mice), and GWI+AV (Gulf War chemical and antiviral-treated mice). All intestine sections were counterstained with DAPI (blue) and 3–10 images were taken from the different microscopic field of each group at 20× magnification. The morphometric analysis (calculated as % ROI of red immunofluorescence image (*n* = 3–10)) of images were plotted as a bar graph as Claudin-2 (**B**). (**C**) mRNA expression of epithelial tight junction proteins ZO1 and Occludin in GWI, GWI+AB and GWI+AV groups. Data points were represented with means ± SEM. Statistical significance was calculated using unpaired *t*-test; * *p* < 0.05, ** *p* < 0.01, *** *p* < 0.001.

In our recent reports, we have shown that gut microbial dysbiosis induced by Gulf War (GW) chemicals exposure led to gut leaching [4,10]. Therefore, we assessed the effects that GW chemicals and subsequent antibacterial (Enrofloxacin and Neomycin) and/or antiviral (Ribavirin) treatments had on tight junction proteins of mouse intestinal epithelia. The mouse exposed with GW chemicals (GWI) showed increased expression of Claudin-2 as compared to control groups (Figure 4A,B, *p* < 0.001). Interestingly, similar mouse model co-exposed with GW chemicals and antibiotics (GWI+AB) or antiviral (GWI+AV) showed significantly decreased Claudin-2 expression (similar to control) as compared to GWI group (Figure 4A,B, *p* < 0.001). Similarly, mRNA expression of ZO1 and Occludin (tight junction genes) showed a significant decrease in GWI group of mice as compared to control (Figure 4C, *p* = 0.03 and *p* = 0.023). Interestingly, antibiotic treatment (GWI+AB) recovers both ZO1 and Occludin gene expression as compared to GWI groups (Figure 4C, *p* = 0.02 and *p* = 0.026). Similarly, antiviral treatment (GWI+AV) significantly improves both ZO1 and Occludin gene expression as compared to GWI groups (Figure 4C, *p* = 0.008 and *p* = 0.03). These data suggest that while antibiotic treatment significantly altered the bacterial dysbiosis and antiviral treatment for gut partial virome depletion significantly improved viral dysbiosis induced by GW chemical exposure, these treatments also restored tight junction protein levels along the intestinal epithelial barrier.

### 3.4. Viral Dysbiosis in GWI Mice Was Accompanied by Intestinal Inflammation in GWI

**Figure 5:**
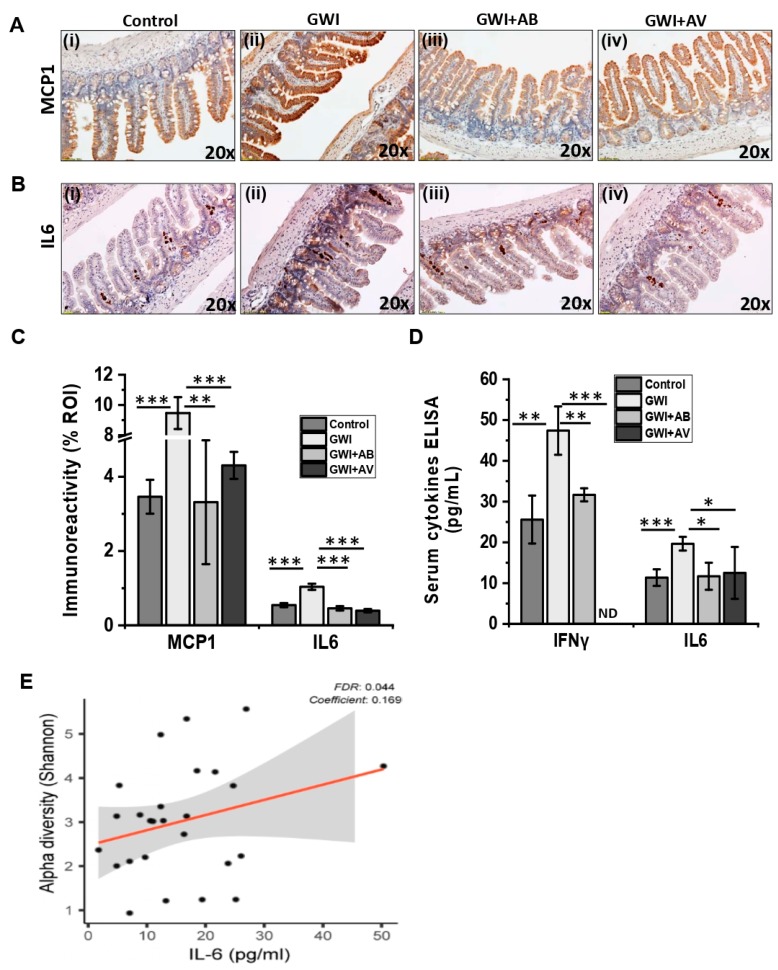
Anti-viral or antibiotic intervention ameliorates intestinal inflammation in GWI. Representative immunohistochemistry images of small intestine showed immunoreactivity of MCP1 (**A**) and IL6 (**B**) in control, GWI (Gulf war chemical exposed mice), GWI+AB (Gulf War chemical and antibiotic exposed mice), and GWI+AV (Gulf War chemical and antiviral-treated mice). 3–10 images were taken from the different microscopic field of each group at 20× magnification. (**C**) The morphometric analysis (calculated as % ROI) of images were plotted as a bar graph. (**D**) Serum IFNγ and IL6 levels in pg/mL were plotted as a bar graph. IFNγ levels in GWI+AV group were below the detection limit and represented as ND (not detectable). For both (**C**) and (**D**) data points were represented with means ± SEM. Statistical significance was calculated using unpaired *t*-test; * *p* < 0.05, ** *p* < 0.01, *** *p* < 0.001. (**E**) Correlation plot of virome Shannon diversity index by serum IL-6. Results of multivariate analyses for association with serum ELISA measurements are shown. Linear regression is shown in red, 95% confidence bands are indicated in gray. False discovery rate (FDR), FDR adjusted q-value; Coefficient, regression coefficient.

Gut microbial homeostasis regulates innate and adaptive immune response [17]. Recent studies suggest that viral and bacterial dysbiosis is directly associated with intestinal inflammatory disease such as IBD, Crohn’s disease, and ulcerative colitis [23,26,42,43]. In this study, we observed significant dysbiosis in both virome and bacteriome in mice exposed to GW chemicals. Based on such rationale, we assessed the association of gut virome dysbiosis in intestinal inflammation. We found that the mice exposed with GW Chemicals (GWI group) that had an altered virome diversity compared to controls showed a significant increase in monocyte chemoattractant protein 1 (MCP1) and interleukin 6 (IL6) as compared to control mice group (Figure 5Ai,ii,Bi,ii,C, *p* < 0.001). Interestingly, mice co-exposed with GW chemicals and antibiotic (GWI+AB) showed a significant decrease in both MCP1 and IL6 immunoreactivity as compared to GWI groups of mice (Figure 5Aii,iii,Bii,iii,C; *p* < 0.006). Similarly, antiviral treatment to the mice exposed with GW chemical (GWI+AV) showed a significant decrease in both MCP1 and IL6 immunoreactivity as compared to GWI groups of mice (Figure 5Aii,iv,Bii,iv,C; *p* < 0.001). We also studied whether the GW chemical exposure to the mice imposed any systemic inflammation. Results showed that the GW chemical-exposed mice (GWI) that had virome dysbiosis was strongly associated with a significant increase in serum IFNγ and IL6 levels as compared to control mice (Figure 5D; *p* < 0.002). Interestingly, antibiotic treatment (GWI+AB) showed reduced serum IFNγ and IL6 levels as compared to the GWI mice (Figure 5D; *p* < 0.017). Similarly, the antiviral-treated group (GWI+AV) also showed significantly decreased serum IFNγ and IL6 levels as compared to GWI mice (Figure 5D; *p* < 0.03). Since GWI disease pathology was associated with an elevated inflammatory profile, we examined the effects of inflammation on the virome. Indeed, lower levels of serum proinflammatory cytokine IL6 was associated with a lower virome diversity (i.e., control or antiviral treatment) (Figure 5E). Conversely, elevated levels of serum proinflammatory cytokine IL6 was associated with a higher virome diversity. Serum IFN-γ and IL-1β also positively correlated with increased virome diversity found in GWI (Supplementary Figure S2).

### 3.5. Altered Resident Enteric Virome Signature Was Accompanied by Activated TLR Signaling in Intestinal Inflammatory Phenotype of GWI

**Figure 6:**
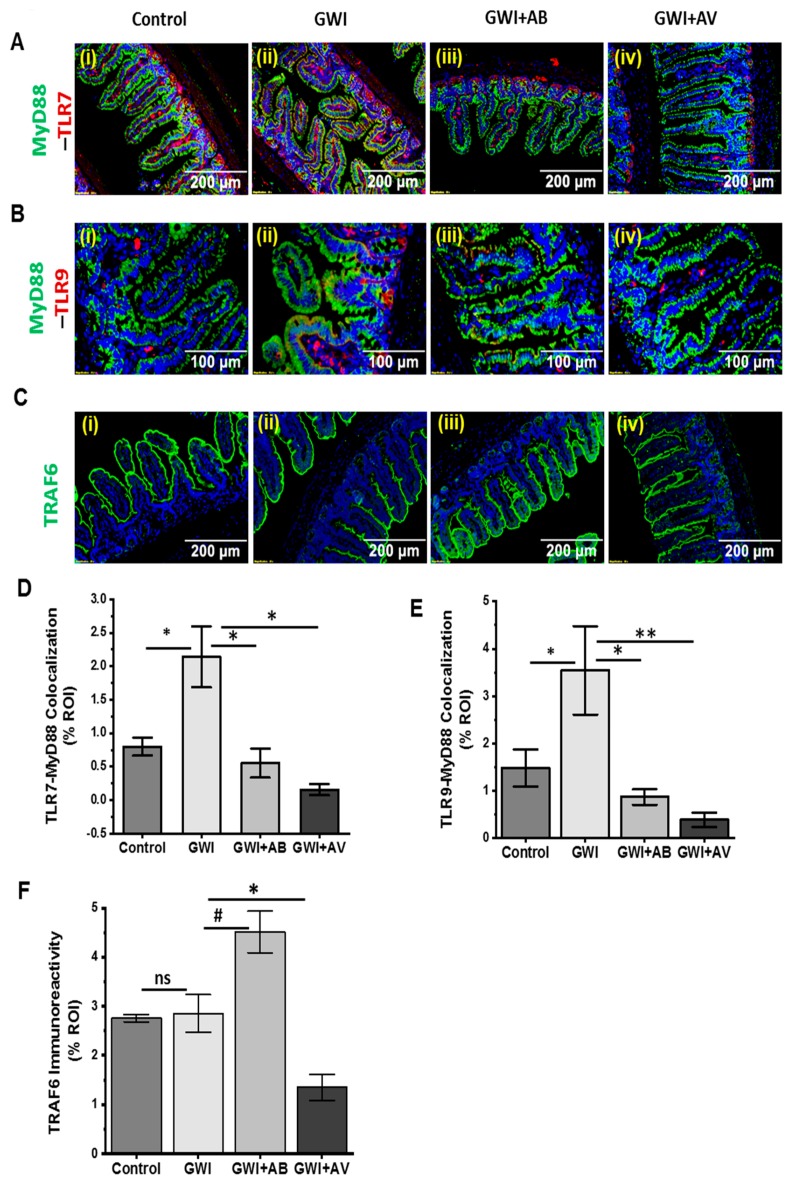

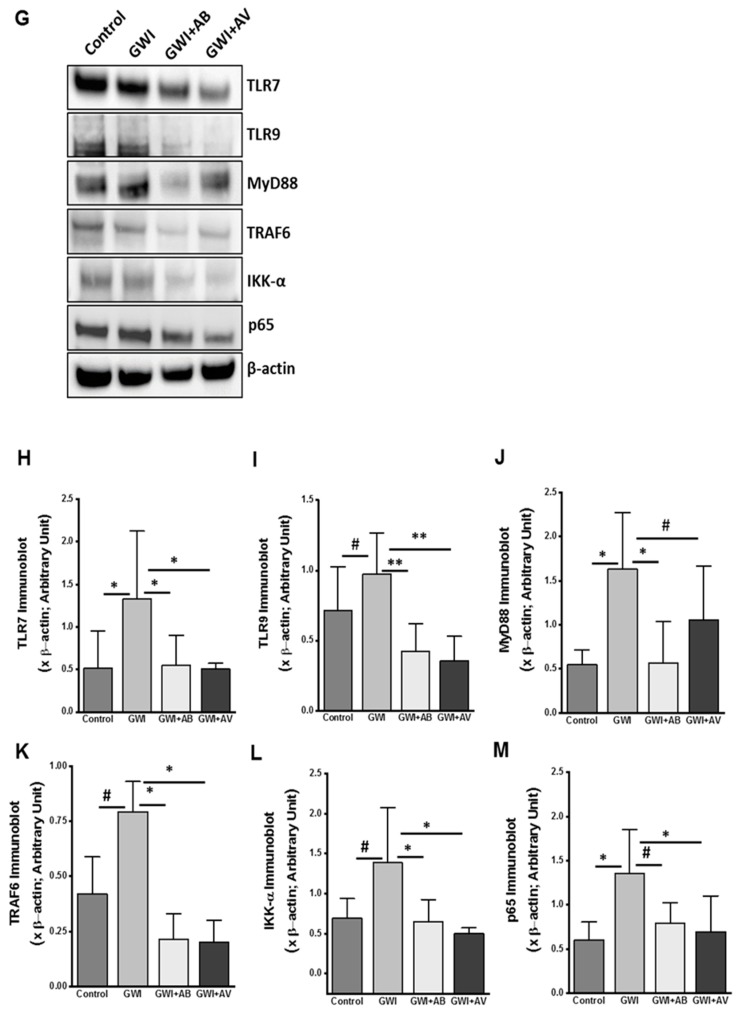
Gut viral dysbiosis activates TLR7 and TLR9 pathway in GWI. Representative immunofluorescence images of small intestine showed (**A**) TLR7 (red) and MyD88 (green); (**B**) TLR9 (red) and MyD88 (green) and (**C**) TRAF6 (green) immunoreactivity in Control, GWI (Gulf war chemical exposed mice), GWI+AB (Gulf War chemical and antibiotic exposed mice), and GWI+AV (Gulf War Chemical and antiviral-treated mice). All intestine sections were counterstained with DAPI (blue) and 3–15 images were taken from the different microscopic field of each group at 20× or 40× analysis of colocalized area (yellow, calculated as % ROI) of TLR7-MyD88 (**D**), TLR9-MyD88 (**E**), and immunoreactivity of TRAF6 (**F**) was plotted as a bar graph. (**G**) Western blot analysis of TLR7 and TLR9, and its signaling cascade molecules such as MyD88, TRAF6, IKK-α, and p65 were plotted as immunoblot. (**H**–**M**) Morphometry analysis of all immunoblot (*n* = 3–8) normalized against β-actin was plotted as a bar graph. Data points were represented with means ± SEM. Statistical significance was calculated using unpaired *t*-test; ^#^ *p* < 0.1, * *p* < 0.05, ** *p* < 0.01. ns = non-significant at *p* ≥ 0.1.

In recent studies, enteric viruses have been identified to alter the innate immune response in several diseases [44,45]. Because enteric viruses interact with Toll-like receptors (TLRs: e.g., TLR3, TLR7, TLR9) to activate pro-inflammatory or anti-inflammatory signaling in intestinal inflammation, we investigated the role of gut viral dysbiosis in alteration of the innate immune system in GWI. We observed that viral dysbiosis in GW chemical-exposed group (GWI) activates TLR7 and TLR9 signaling pathway. GW chemical exposure that had altered virome signature showed significant increased level of TLR7 and TLR9 colocalization with MyD88 adapter molecule (*p* < 0.05, *n* = 15)), a likely event of TLR activation as compared to the control group (Figure 6Ai,ii,Bi,ii,D,E). However, the antiviral or antibiotic intervention significantly decreased the TLR7 and TLR9 expression (red) and colocalization with MyD88 (Figure 6Aii–iv,Bii–iv,D,E; *p* < 0.03). Further, TNF Receptor Associated Factor 6 (TRAF6), that plays a significant role in TLRs signaling cascade was studied. The immunoreactivity of TRAF6 showed an increase in GWI+AB compared to GWI (Figure 6Ci–iii,F; *p* > 0.061) while the same was unchanged when compared between controls and GWI mice. However, antiviral-treated mice (GWI+AV) showed a significantly decreased immunoreactivity as compared to GWI mice (Figure 6Cii–iv,F; *p* < 0.04). Next, to investigate total protein expression of TLRs signaling cascade, western blot of TLR signaling molecules were carried out in intestinal protein lysate. Results showed that the expression of TLR7, MyD88, and p65 were significantly increased in GWI groups that had altered virome diversity as compared to control groups (Figure 6G,H,J,M; P < 0.029; *n* = 5). Importantly, TLR9, IKK-α, and TRAF6 showed a increase in GWI groups as compared to control group (Figure 6G,I,K,L; *p* < 0.06; *n* = 5). Interestingly, expression of these cascade molecules in GWI+AB groups and GWI+AV groups showed a significant decrease in TLR7, TLR9, MyD88, TRAF6, and IKK-α in GWI+AB groups (*p* < 0.01) and TLR7, TLR9, TRAF6, IKK-α, and p65 in GWI+AV groups (*p* < 0.030) respectively (Figure 6G–M; *n* = 5). We also observed a non-significant decrease inp65 in GWI+AB groups (*p* = 0.08) and MyD88 in GWI+AV groups (*p* = 0.09) respectively (Figure 6G–M, *n* = 5).

A recent study suggests that dsRNA viruses activate TLR3 pathways resulting in interferon-beta (IFNβ) production [46]. In another study, IFNβ is shown to have antiviral and anti-inflammatory properties [47]. Based on the above rationale, we investigated the TLR3 mediated anti-inflammatory response in this study. Results showed a significant increase of TLR3 expression in GWI group as compared to control group (*p* < 0.05) and a significant decrease in GWI+AB or GWI+AV groups as compared to GWI group (*p* < 0.05) (Supplementary Figure S3A–D). Similarly, a nonsignificant increase in serum IFN-β was observed in GWI group as compared to control. Also, GWI+AB group showed a nonsignificant decrease as compared to GWI group (Supplementary Figure S3E). However, antiviral-treated group (GWI+AV) showed a significant decrease in serum IFN-β as compared to GWI group (Supplementary Figure S3E). The above data suggested that enteric viral dysbiosis primarily activated TLR7 and TLR9 mediated proinflammatory response in GWI while TLR3 mediated pathways might be an adaptive response from the host. Also, treatment with Ribavirin (broad-spectrum antiviral) downregulated TLR3 and TLR7 pathway and thus played a significant role in intestinal inflammation in GWI that also had viral alterations. 

### 3.6. Inhibition of Virome Alteration by Ribavirin in GWI Decreased TLR4 Activation but Had Limited Impact on TLR4 Protein Levels

**Figure 7:**
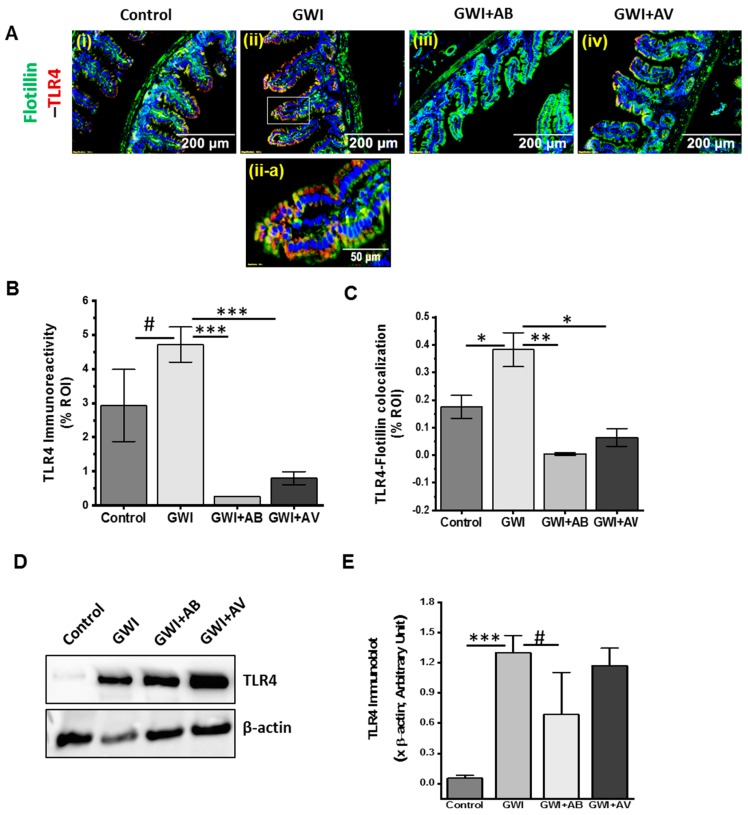
Gut bacterial dysbiosis activates TLR4 pathway in GWI. (**A**) Representative immunofluorescence images of small intestine showed TLR4 (red) and flotillin (green, lipid raft) immunoreactivity in Control, GWI (Gulf war chemical exposed mice), GWI+AB (Gulf War chemical and antibiotic exposed mice), and GWI+AV (Gulf War Chemical and antiviral-treated mice). All sections were counterstained with DAPI (blue) and 3–15 images were taken from different microscopic field of each group at 20× magnification. (**A-iia**) 3× digital zoom of boxed area of image A-ii showing colocalization events. (**B**) The morphometric analysis of TLR4 (red) immunoreactivity calculated as % ROI were plotted as bar graph. (**C**) The morphometric analysis of TLR4 (red)- Flotillin (green) colocalization (yellow) events calculated as % ROI were plotted as bar graph. (**D**) Western blot analysis of TLR4 and β-actin protein in small intestine of Control, GWI, GWI+AB, and GWI+AV. (**E**) Morphometry analysis of TLR4 immunoblot (*n* = 5) normalized against β-actin was plotted as a bar graph. Data points were represented with means ± SEM. Statistical significance was calculated using unpaired *t*-test; ^#^ *p* < 0.1, * *p* < 0.05, ** *p* < 0.01, *** *p* < 0.001.

We and others have shown that the TLR4 is a prime innate immune molecule, activated by DAMPs and PAMPs in Gulf war illness [4,10]. Consistent with our previous finding, results in this study showed higher TLR4-flotillin immunoreactivity in GWI groups as compared to control groups (Figure 7Ai,ii,B; *p* < 0.1). Also, a significant increase in TLR4 protein was observed in immunoblot analysis (Figure 7D,E; *p* < 0.001). On the other hand, antibiotic-treated (GWI+AB) and antiviral-treated (GWI+AV) groups showed a significant decrease in the TLR4-Flotillin colocalization (Figure 7Aii–iv,B,C; *p* < 0.008) but did not impact TLR4 protein levels (Figure 7D,E).

### 3.7. Altered Gut Virome and Microbiome in Gwi Mice Was Associated with Blood–Brain Barrier (BBB) Integrity Loss and Neuroinflammation While Antiviral Ribavirin Reversed Outcomes

**Figure 8:**
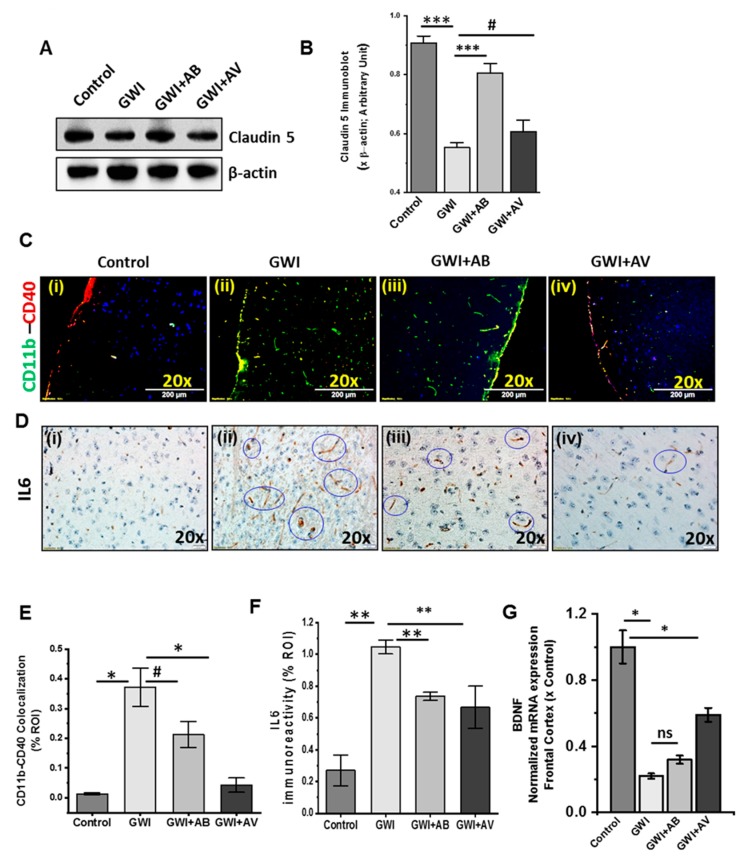
Anti-viral or antibiotic intervention improves neuroinflammation in GWI. (**A**) Western blot analysis of Claudin-5 and β-actin protein in the frontal cortex of Control, GWI (Gulf war chemical exposed mice), GWI+AB (Gulf War chemical and antibiotic-treated mice), and GWI+AV (Gulf War Chemical and antiviral-treated mice). (**B**) Morphometry analysis of Claudin-5 immunoblot (*n* = 5) normalized against β-actin were plotted as a bar graph. (**C**) Representative immunofluorescence images of frontal cortex showed CD40 (red) and CD11b (green) immunoreactivity and colocalization (yellow, marked with the arrows) in Control, GWI, GWI+AB, and GWI+AV mice. All sections were counterstained with DAPI (blue) and 3–15 images were taken from the different microscopic field of each group at 20X magnification. (**D**) Immunohistochemistry images for IL6 expression in the frontal cortex of Control, GWI, GWI+AB, GWI+AV. The prominent immunoreactivities were marked with the blue circles. (**E**) The morphometric analysis of colocalized area (yellow, calculated as % ROI) of CD11b-CD40 was plotted as a bar graph. (**F**) Morphometric analysis of IL6 immunohistochemistry images (*n* = 3–15) calculated at % ROI. (**G**) mRNA expression of BDNF in mouse frontal cortex of Control, GWI, GWI+AB, and GWI+AV. The RT-PCR data were normalized against internal control 18S and plotted as fold-change to the control. Data points were represented with means ± SEM. Statistical significance was calculated using unpaired *t*-test; ^#^ *p* < 0.10, * *p* < 0.05, ** *p* < 0.01, *** *p* < 0.001. ns = non-significant at *p* ≥ 0.1.

We and others have shown that GW chemical exposure led to neuroinflammation [4,10]. A healthy BBB plays a gatekeeper role in protecting the brain from unwanted circulatory agents which might be harmful to the brain. Therefore, in this study, we investigated the integrity of BBB and microglial activation leading to neuroinflammation. Results showed that Claudin-5 protein (brain endothelial tight junction protein) expression significantly decreased in GWI mice as compared to control in western blot analysis (Figure 8A,B; *p* < 0.001). Interestingly, GWI+AB group showed significant improvement in Claudin-5 as compared to GWI (Figure 8A,B; *p* < 0.001) and antiviral treatment (GWI+AV) also showed higher expression as compared to GWI but was not significant (Figure 8A,B; *p* = 0.097). Next, we investigated cortical microglia activation by using both the resting stage marker (CD11b) and active stage marker (CD40). Immunofluorescence imaging showed that a significant higher immunoreactivity and colocalization of both CD11b and CD40 marker in GWI group as compared to control group (Figure 8Ci,ii,E; *p* = 0.005). However, GWI+AB group showed a nonsignificant decrease as compared to GWI (Figure 8Cii,iii,E; *p* = 0.064) while antiviral treatment (GWI+AV) showed a significant decrease in CD11b–CD40 expression and colocalization as compared to GWI (Figure 8Cii–iv,E; *p* = 0.019). Further, we tested mice for the expression of pro-inflammatory cytokine IL6 in the frontal cortex. Immunohistochemistry results showed a significant increase in IL6 immunoreactivity (blue circled) in GWI groups as compared to control (Figure 8Di,ii, F; *p* < 0.002). Interestingly, both the GWI+AB group and the anti-viral treated group showed a significant decrease in IL6 immunoreactivity as compared to GWI (Figure 8Dii–iv,F; *p* < 0.004). Next, we examined the mRNA expression of Brain-Derived Neurotrophic Factor (BDNF; regulate neuronal health) in the frontal cortex. We found a significant low level of BDNF mRNA expression in GWI as compared to control (*p* = 0.02) and GWI+AB group showed a nonsignificant increases BDNF levels. Interestingly, GWI+AV group showed significant increase in BDNF expression as compared to GWI (Figure 8G, *p* = 0.19 and 0.04 respectively). These data suggested that the alteration in gut virome and microbiota induced intestinal and systemic inflammation led to BBB integrity loss, microglial activation, and neuroinflammation in GWI. Treatment with antibiotic or antiviral compound improved outcomes. Interestingly, BDNF, a neurogenesis marker was negatively correlated with GWI virome diversity (Supplementary Figure S2).

## 4. Discussion

The well-characterized GWI symptoms are GI inflammatory disease, chronic fatigue, neuroinflammation, and cognitive impairment [48]. The current study investigates a mechanistic analysis of the gut virome-bacteriome mediated GI and neuroinflammation. To our knowledge, this is the first study that examined GW chemical-induced virome dysbiosis and bacteriome-virome interactions in GWI. In our previous study, we have shown that GW chemical exposure causes bacterial dysbiosis and that led to the loss of healthy gut bacteria such as Lactobacillus and Bifidobacterium [4]. Gut dysbiosis induced leaky gut symptoms and systemic inflammation in GWI [4]. In a recent review, it has been reported that gut virome is the missing link between bacterial dysbiosis and host immunity [11]. The same study also described the role of virome in TLR signaling and GI diseases and hence concluding that virome analysis may lead to a novel therapeutic strategy for GI complications [11,17,49]. The above study prompted us to explore the role of enteric viruses in GWI pathology and as a probable therapeutic target. In the present study, we found that the GW chemical exposure led to an increase in virome richness and relative abundance of dsDNA bacteriophages *Myoviridae*, *Siphoviridae*, and *Caudovirales* and decreased relative abundance of ssDNA bacteriophages *Microviridae*. We used a broad-spectrum antiviral compound Ribavirin to test the hypothesis that (a) inhibition of gut viruses in the host may prove the role of gut virome in GWI and (b) antiviral treatment may help to reconstruct and restore the virome signature in GWI. Ribavirin has been extensively used as a therapeutic drug in several viral infections including Paramyxovirus, Adenovirus, and RSV infections [50,51,52]. Our results showed that Ribavirin reverses GWI-induced alterations of the enteric viral community. The enteric bacteriophage-bacteria (parasite-host) interaction is an essential phenomenon to regulate both bacterial and viral community in the gut and play as a key role in gut health [53]. Here, we found an independent or cumulative effect of both virome and bacteriome dysbiosis in weakening of GI tight junctions (increased Claudin-2 and decreased ZO1 and Occludin), GI inflammation (increased MCP1 and IL6), systemic inflammation (increased serum IFNγ and IL6) and neuroinflammation (increased microglia activation and brain IL6). The present study also showed that Ribavirin treatment improved GI tight junction assembly and subsequently decreasing GI and neuroinflammation. Interestingly, inducing gut sterility by using antibiotics also improves such GI and neurological complications. Results reported here are also strongly supported by previous such studies where Ribavirin or antibiotics were applied as therapeutics in other GI complications and neurological illnesses [15,54,55,56,57]. However, nonavailability of germ-free and gnotobiotic mice or mice completely devoid of gut viruses prove to be a serious handicap in establishing the exact role of viruses in GWI or any other disease pathology though use of antiviral cocktails similar to our approach have been shown to serve the purpose. 

Gut microbiome is a collection of trillions of microorganisms that modulated host pathology and physiology through different mechanisms. Previous reports have suggested that the intestinal microbiota had a strong influence on the mucosal immune system and vice versa [58]. A balanced pathogen and commensal microbiome composition maintain the microbiome-host immune homeostasis. However, any alteration in the microbiome may activate innate and adaptive immune responses and may progress to an inflammatory phenotype [17,26,58,59,60]. In the present study we have shown that the GW chemical-induced virome richness and dysbiosis showed a strong association with activated Toll-like receptor-7 (TLR7) and TLR9 mediated proinflammatory pathways. We also found that the expression and activation of downstream signaling molecules including TLR7-MyD88 (binding of TLR7 with adaptor molecule myosin D88), TLR9-MyD88, or TLR4-Flottilin (localization of TLR4 in lipid raft) complex formation, TRAF6, IKK-α, and p65 (NFκB activation) have been increased upon gut virome and microbiome dysbiosis. The dysbiosis was also accompanied by increased inflammation while use of Ribavirin or antibiotics independently downregulated these pathways and subsequently inflammation. The above results prompted us to study whether virome diversity had a direct connection to a sustained systemic inflammatory response via increased serum IL6 or vice versa. We found that IL6 and virome diversity had a positive correlation in GWI. Studies have shown that the enteric viruses can activate TLR3 signaling pathway to induce anti-inflammatory response via IFNβ production [61,62]. However, it has always been a conflict whether viral-induced TLR3 activation leads to pro- or anti-inflammatory response in viral pathogenesis [45,63,64]. In our study, we observed that increased TLR3 might switch to pro-inflammatory response via IL6 or fail to produce significant IFNβ in order to support its anti-inflammatory role in GWI though such mechanisms in the host remain inconclusive at this time. 

Another significant area of GWI pathology concerns neurological illness which includes neuroinflammation, cognitive impairment, neurodegeneration and memory loss [48,65,66]. The blood–brain barrier (BBB) is selective and prevents harmful mediators such as reactive oxygen species (ROS), inflammatory cytokines, pathogen-associated molecular patterns (PAMPs), and damage-associated molecular patterns (DAMPs) to reach the brain [67,68]. Reports suggest that in several microbial infections, the integrity of blood–brain endothelial cytoskeleton and tight junctions are likely to be compromised [69]. Similarly, stress along with systemic inflammation (such as in GWI) can increase BBB permeability [70,71]. Our results showed decreased levels of Claudin-5 (tight junction protein of BBB) in GWI which is prevented upon antibiotic treatment but was not significantly different with the antiviral treatment though the virome alterations in the gut was strongly associated with low Claudin-5. This compromised BBB that was reflected by decreased Claudin-5 in our model might be caused by a leaky gut and systemic inflammation. This hypothesis has been supported by another study that discussed leaky gut and leaky brain in celiac disease [72]. Also, an evidence of circulatory IL6 modulating blood–brain barrier function in the ovine fetus seems to support our present finding where we find higher circulatory IL6 and decreased Claudin-5 [73]. An altered virome in our GWI mouse model and subsequent restoration of virome diversity similar to controls (healthy) by Ribavirin treatment and its relationship with IL6 and Claudin-5 levels show a strong correlation of host viruses likely playing a central role in systemic and neuroinflammation. Therefore, we can justifiably assume that altered virome associated leaky gut and systemic inflammation likely contributes to BBB integrity loss/dysfunction though much mechanistic details are yet to emerge. Studies have shown that BBB dysfunction leads to various neurological diseases such as dementia, Alzheimers, autism spectrum disorders, depression, chronic headache and neuroinflammation [72]. Our results of increased microglia activation and proinflammatory cytokine IL6 in the frontal cortex of GWI mice with altered virome signature has great clinical significance in understanding persistent neuroinflammation in GWI veterans. Since activated microglia can secrete IL6, it is likely that the observed increased IL6 may be playing a central role in the neurological symptoms in GWI. The fact that altered virome is strongly correlated with higher circulatory IL6 and microglial activation, the present study may throw important viewpoints for further mechanistic studies into cognitive impairment associated with GWI. 

Limitations of the study: GWI is a complex neuroimmune disease and modelling the disease in mice is always a challenge. Our model, though has established its closeness to the human disease is not perfect. The results reported here needs to evolve and future studies should test the results in multiple rodent models that currently exist. Administration of Gulf war chemicals such as permethrin, DEET or other organophosphates should be tested for application through the dermal routes to study whether the virome or bacteriome diversity still occurs due to change of the route of administration. Secondly, the models used routinely are acute in nature that tests the alterations immediately after the administration of chemicals are concluded. The approach should take into consideration the time lag between the war and the present day to suitably model the current day veteran. Persistence of the bacteriome and virome diversity in GWI should be tested in persistence models of GWI to better obtain a likely scenario of today’s Gulf War veterans. This might help in finding an effective cure for our veterans.

In summary, we present first direct evidence that Gulf War theater chemicals might increase host gut virome richness, and cause both virome and bacteriome dysbiosis. Further, altered virome associated gut membrane integrity loss probably activated the innate immune response in the intestine via virome particle-induced activation of TLR7 and TLR9. The viral diversity in GWI via IL6 may cause BBB dysfunction and neuroinflammation. The study also presents evidence that the use of antivirals like Ribavirin or along with the combinations of complementary and alternative compounds and probiotics might restore gut biome homeostasis and can form the basis of a novel treatment strategy for GWI and similar disorders.

## Figures and Tables

**Table 1:** The sequences for the primers used for real-time PCR.

Gene	Sequences (3′-5′)
ZO1	Sense: CCACCTCTGTCCAGCTCTTC Antisense: CACCGGAGTGATGGTTTTCT
Occludin	Sense: GTGAGCTGTGATGTGTGTTGAGCT Antisense: GTGGGGAACGTGGCCGATATAATG
BDNF	Sense: TGCAGGGGCATAGACAAAAGG Antisense: CTTATGAATCGCCAGCCAATTCTC

## Supplementary Materials

The following are available online at https://www.mdpi.com/1999-4915/11/10/968/s1, Supplementary Figure S1. Gut virome sequencing in the mouse model of Gulf War Illness and controls. (A) Sequencing read depth of samples from the three Illumina MiSeq runs. Statistical significance was assessed by Mann–Whitney U test; ns, not significant. (B) The plot shows viral sequencing reads identified within each treatment group. Statistical significance was assessed by Mann–Whitney U test; ns, not significant. Principal coordinate analysis (PCoA) of unweighted Bray–Curtis distance of virome is shown colored by the Illumina NGS run (left); or by specimen type (right): stool samples (control, GWI, GWI+AB and GWI+AV groups), PBS extraction negative controls. (D) The relative abundance of the 30 most abundant virus species is shown of PBS extraction negative controls and stool samples, per Illumina run. (E) Microviridae family and (F) Caudovirales order abundance, (G–H) Principal coordinate analysis (PC1, PC2 and PC1-PC2) of binary Jaccard distance of virome species, (I) PCoA plot of virome unweighted Bray-Curtis distance. 95% confidence intervals of each group are shaded accordingly. (J) Rarefaction curves of bacterial richness (species) and (K) bacterial species richness of each group is plotted. (L) Viral contig richness and (M) Shannon diversity index of viral contigs for each group is shown. (N) PCoA analyses of binary Jaccard distance of viral contigs is shown. 95% confidence intervals of each group are shaded accordingly. (O) Heatmap shows Pearson correlations of Bacteriophage contigs (columns) and bacteria (rows). Clustering was performed on the control group (left) and the same clustering order maintained in correlation plots for GWI, GWI+AB and GWI+AV groups. Supplementary Figure S2. Pearson’s correlation analysis of Virome alpha diversity and gene expression of tight junctions and inflammatory markers. Correlation plot of virome alpha diversity by serum (A) IL-1β (pg/mL), (B) IFN-γ (pg/mL) and (C) IL-6 (pg/mL), and gene (mRNA) expression of (D) IFNγ, (E) TNF-α, (F) ZO1, (G) Claudin-2 and (H) BDNF. Results of multivariate analyses for association measurements are shown in table and linear regression is shown in red. Supplementary Figure S3. Gut viral dysbiosis activates TLR3 pathway but did not show a protective role in GWI via IFN β. Representative immunofluorescence images of small intestine showed TLR3 (red) and β-tubulin (green) immunoreactivity in Control, GWI (Gulf war chemical exposed mice), GWI+AB (Gulf War chemical and antibiotic exposed mice), and GWI+AV (Gulf War Chemical and antiviral-treated mice). All sections were counterstained with DAPI (blue) and 3–15 images were taken from the different microscopic field of each group at 20× magnification. (B) The morphometric analysis of TLR3 immunoreactivity calculated as % ROI were plotted as a bar graph. (C) Western blot analysis of TLR3 and β-actin protein in the small intestine of Control, GWI, GWI+AB, and GWI+AV. (D) Morphometry analysis of TLR3 immunoblot (*n* = 5) normalized against β-actin was plotted as a bar graph. (E) Serum level of IFNβ (U/mL) in Control, GWI, GWI+AB, GWI+AV. Data points were represented with means ± SEM. Statistical significance was calculated using unpaired *t*-test; ^#^ *p* < 0.1, * *p* < 0.05, ** *p* < 0.01, *** *p* < 0.001. ns = non-significant at *p* ≥ 0.1.
