# Supplementary material for: Gut DNA Virome Diversity and Its Association with Host Bacteria Regulate Inflammatory Phenotype and Neuronal Immunotoxicity in Experimental Gulf War Illness
Source: Viruses. 2019 Oct 21;11(10):968. doi: 10.3390/v11100968 (PMC6832151; doi:10.3390/v11100968)
Supplement: Supplementary file 1 [file viruses-11-00968-s001.zip › viruses-599965-supplementary.pptx]

## Slide 1
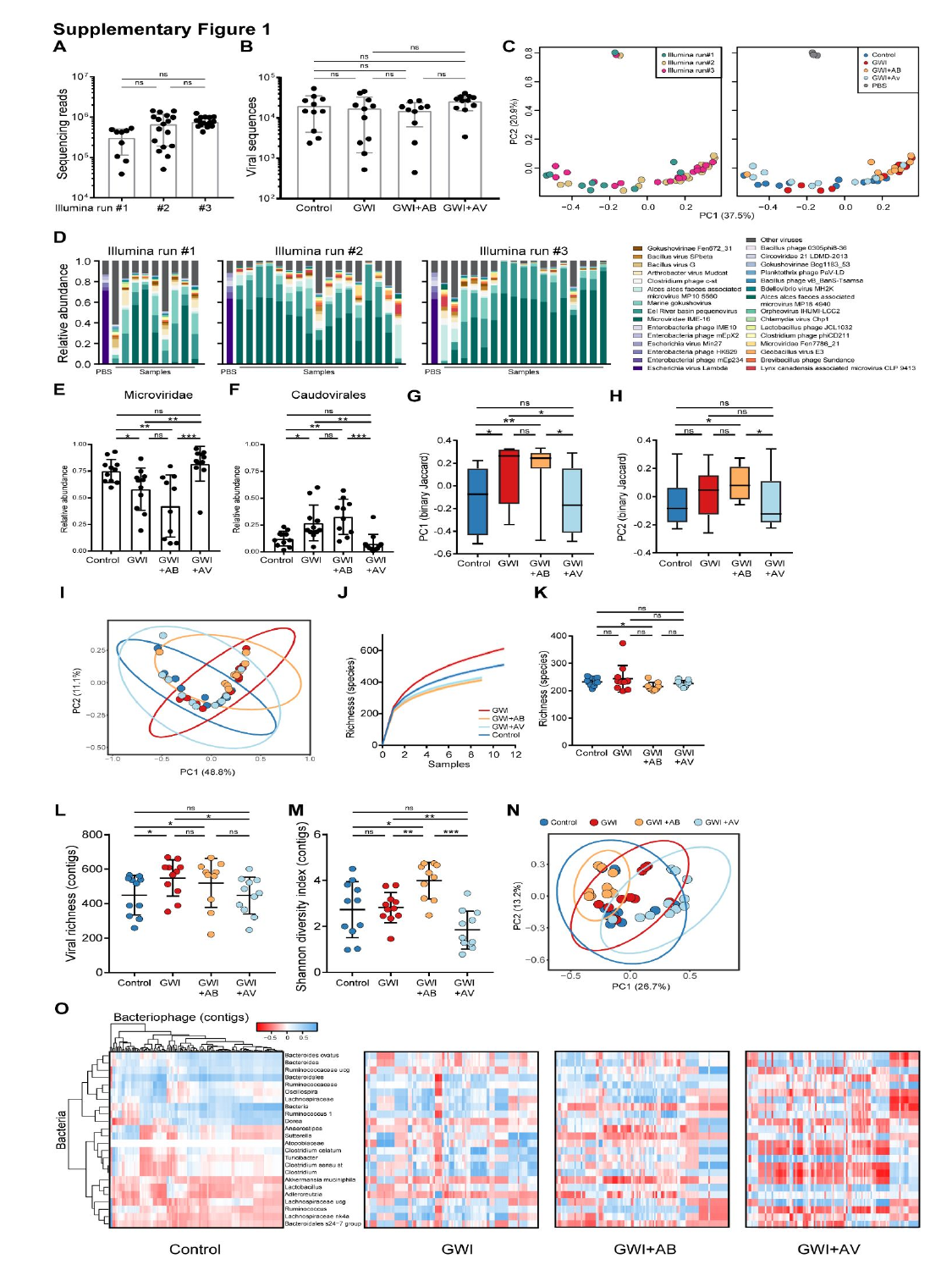

## Slide 2
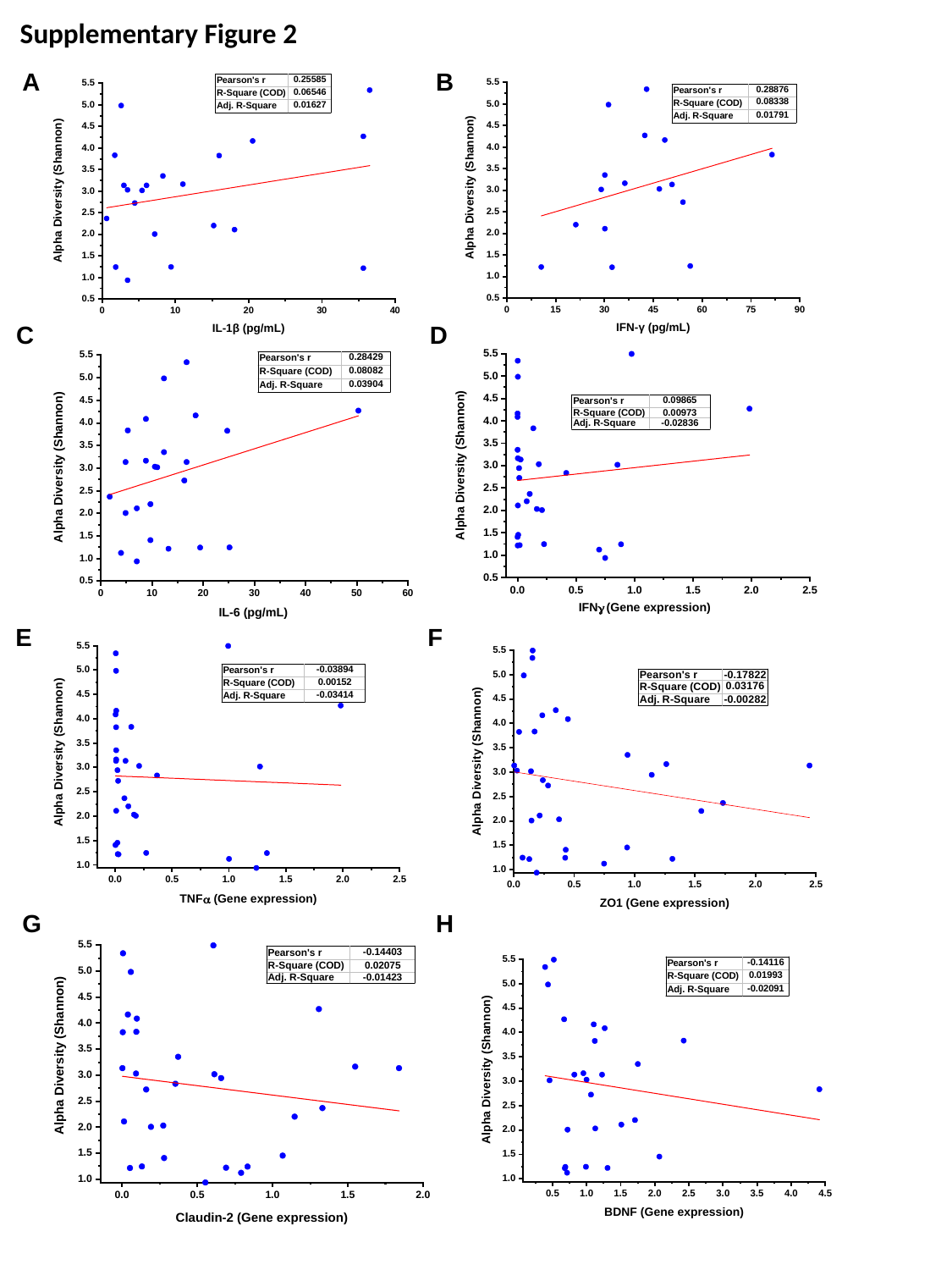

Supplementary Figure 2
A
B
C
D
E
F
G
H

## Slide 3
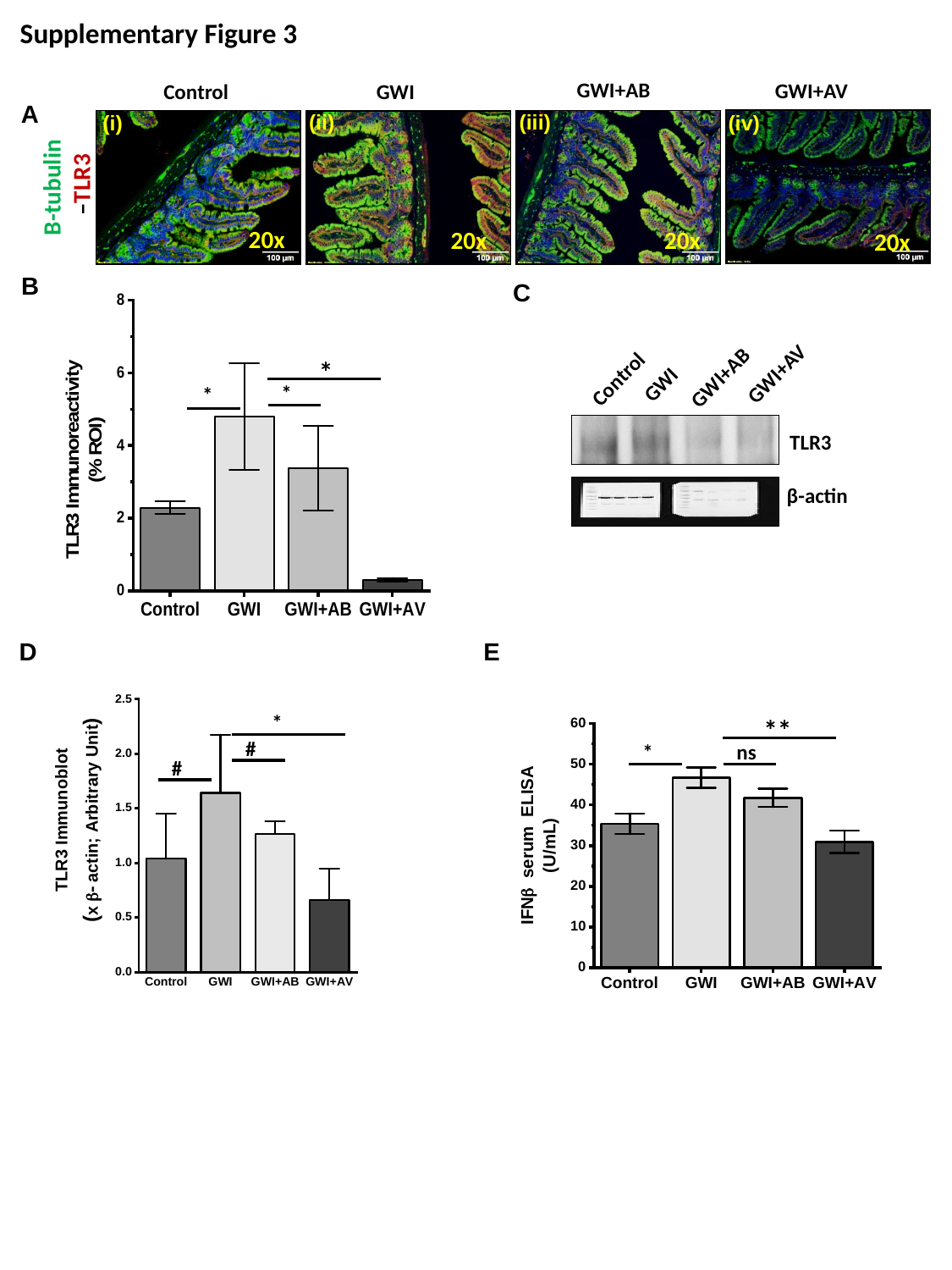

Supplementary Figure 3
GWI+AB
GWI+AV
Control
GWI
A
(ii)
(iii)
(i)
(iv)
Β-tubulin
–TLR3
20x
20x
20x
20x
B
C
GWI+AV
GWI+AB
Control
GWI
TLR3
β-actin
*
*
*
E
D
*
**
#
*
ns
#
